# Comparison of Physical Activity Levels in Youths before and during a Pandemic Lockdown

**DOI:** 10.3390/ijerph18105139

**Published:** 2021-05-12

**Authors:** Małgorzata Bronikowska, Jana Krzysztoszek, Marlena Łopatka, Mateusz Ludwiczak, Beata Pluta

**Affiliations:** 1 Department of Recreation, Poznan University of Physical Education, 61-871 Poznań, Poland; ludwiczak@awf.poznan.pl (M.L.); bpluta@awf.poznan.pl (B.P.); 2 Department of Didactics of Physical Activity, Poznan University of Physical Education, 61-871 Poznań, Poland; krzysztoszek@awf.poznan.pl (J.K.); lopatka@awf.poznan.pl (M.Ł.)

**Keywords:** pandemic, lockdown, physical (in) activity, adolescents

## Abstract

This study aimed to compare physical activity (PA) levels before and during a pandemic lockdown among adolescent Polish youths in relation to meeting the World Health Organization’s (WHO) recommendations of moderate-to-vigorous physical activity (MVPA) and identify potential environmental factors that may affect these levels. An online survey tool that included validated measures of adolescents’ MVPA and socio-environmental, potential confounders was administered to youths (e.g., lack of classmates, motivation, possibility of choosing any physical exercise time, possibility of choosing your own exercises, home environment). The analytic sample comprised 127 adolescents aged 15.4 ± 0.5 years on average (52% girls and 48% boys). The results highlight that among adolescents who did not meet the WHO MVPA recommendations before the lockdown, 13.4% had an increased frequency of PA (from 2.9 to 5.4 day/week) (*p* = 0.01) during the lockdown. In the group who did meet these recommendations before the lockdown, 50% significantly decreased their level of MVPA below the WHO’s recommendations (*p* = 0.01). Self-rated health for PA showed a significantly strong correlation with MVPA in the group of students who met the WHO recommendations before the pandemic (r = 0.76; *p* < 0.05) but failed to maintain that recommendation during the COVID-19 restriction. The results suggest a negative impact of the pandemic on PA undertaken by youths. Based on these results we see the need for increased action by both Physical Education (PE) teachers and parents to motivate and support youngsters in taking up systematic PA consciously.

## 1. Introduction

The SARS-CoV-2 pandemic has become an emerging, rapidly evolving state globally, irrespective of socio-economical or cultural development status. The present scenario of the COVID-19 pandemic points to the deepening problem of PA deficiency and indicates a strong relationship with non-communicable diseases (NCDs) [1]. The evidence suggests that obesity, diabetes, and cardiovascular disease are additional risk factors in the complications of COVID-19 [1]. Unfortunately, the pandemic lockdown within governmental restrictions imposed on citizens is not motivating; in fact, it is quite frustrating, specifically regarding the physically active part of the population who has been forced to reduce their daily PA due to these restrictions [2]. Furthermore, so far, no generic guidelines have been issued for promoting PA in a pandemic-related environment in Poland. It seems that well-being, as one of the main aims of public health and the state of health of future generations, is no longer the ultimate public health long-term goal, with concerns of all sorts of governing bodies causing only temporary actions and mending of the situation via restrictions and limitations.

PA is defined as any bodily movement produced by the contraction of skeletal muscles and is an umbrella term that includes subcategories such as sports, leisure time physical activities (LTPAs), dance and physical exercise, which itself is defined as every planned, structured, repetitive, and purposeful intervention [3]. The quarantine requirements, along with social distancing and self-isolation, have perpetuated an environment that promotes physical inactivity and increases the risk of longer-term sedentary behaviours [4]. For children and youths, PA is closely linked with school-related activities, active transport, and sport participation. Since the closure of schools during the COVID-19 pandemic and PE classes becoming inaccessible due to the change to online teaching, this situation could also compromise PA in the population. Lockdown has shifted schoolwork to become even more sedentary than usual [4], despite the evidence that environments promote PA can improve physical, psychological, and mental well-being [5]. For example, exercise has been linked to increased blood flow to the brain and neurotransmitter levels, enhanced plasticity and better focus, attention and information processing in typically-developing children and children with attention-deficit/hyperactivity disorder [6]. In sum, the COVID-19 pandemic has changed the way people approach PA. Due to government-imposed restrictions, outdoors activities have reduced. In the period 1–20 of April, young people under the age of 18 could not leave their home without adult supervision; later they had to keep a distance of 2 m and have their mouth and nose covered. Sports infrastructure was closed until 4 of May [7], with these and other restrictions detrimentally affecting youths’ physical and mental health and potentially leading to chronic ill-functioning conditions and increasing mortality risk [8]. On the other hand, the government’s public health restrictions announcement was aimed at providing more security and safety against the virus’ spread [9], which is an understandable approach given the mortality rate caused by this pandemic.

Therefore, WHO, providing statistics that 81% of adolescents do not get sufficient PA [10], have prepared a new framework and guidelines for promoting health through PA during the COVID-19 pandemic lockdown; this aim has been high on their agenda considering the positive effects of PA and the general recommendations from the second edition of the Physical Activity Guidelines for Americans [11]. These guidelines advocate for at least 150–300 min of moderate intensity or, as a minimum, 75 min of vigorous intensity PA per week (or a comparable arrangement of both) and two sessions of strength training per week. These suggestions agree with Chen’s PA recommendations of at least 30 minutes’ moderate intensity daily and/or at least 20 minutes’ vigorous intensity every other day during the pandemic shutdown [12].

Meeting such recommendations is particularly important for school-age children. A lack of engagement in PA during this human development ontological phase may be irreversible in terms of their sound development [13]. A study by Guddal [14] showed that higher levels of PA are favourably associated with physical health, particularly related to self-esteem, life satisfaction and reduced likelihood of psychological distress for senior high-school students and may assist in their adherence to regular PA participation into adulthood [15]. Kołoło [16] demonstrated the existence of a curvilinear relationship between adolescent PA levels and self-esteem in terms of appearance and body image. Furthermore, several studies suggest that increased PA participation is associated with a more positive body image among adolescent girls [17,18]. This information is of importance when designing PA programmes accessible for children with low physical self-esteem. It is essential to form PA programmes that support and develop sport competence, physical condition, and a sense of body attractiveness among children, especially during a pandemic [10]. Research shows that over 80% of teenagers do not meet the daily PA recommendations of 60 minutes’ MVPA [10,13]. Moreover, MVPA in adolescents declines with age by a mean of 7% per one year [19,20].

It is worth emphasizing that PA is determined by the influence of biological, psychological, social, and environmental factors [21,22,23,24,25,26]. Some studies indicate that a sense of community, which has tended to degrade during the lockdown, is strongly associated with PA engagement levels [27,28,29]. Furthermore, family income and affluence were found to be associated with walking, cycling, and outside playing time among Canadian children during COVID-19 [30]; these authors found that young people living in houses may have easier access to outdoor exercise (e.g., in their backyards) compared to those living in blocks of flats. Even short periods of physical inactivity might improve physical and mental health problems [31]; therefore, walking as a means of transport for providing a daily dose of PA should be promoted. However, with the present situation, all transport forms related to walking are either limited or in decline [32,33,34]. All these issues have deepened the already-poor situation of young people in Poland in the context of their self-esteem and self-rated health/physical fitness. As noted in the latest WHO report [35], Polish adolescents had the highest prevalence of negative body image, with 52% of girls and 31% of boys considering themselves to be too fat, and only 12% of girls and 22% of boys rating their health as excellent. One of the reasons given for this state is the low level of undertaking daily PA at moderate and vigorous (more than 4 days per week) levels, with only 9% of girls and 18% of boys doing so [35,36]. Previous research [37,38,39] shows that PA declines with age, with the steepest decline occurring between the ages of 13 and 18 years. The pandemic situation has deepened this problem [2,10,12,30,36]. Therefore, the authors decided to investigate a section of this reality so that the results of the study could serve future PA professionals (in a research as well as a practical context) to have something to base their PA planning for adolescents in crisis mode on. Thus, there is a global consensus regarding the importance of tracking trends in MVPA and related influential factors that might strongly impact PA among youngsters [40]; additionally, it may be helpful for health and educational authorities in future lockdown situations.

Hence, in this study, we aimed to compare the changes in MVPA that occurred as a result of social distancing during COVID-19 in adolescents from the Wielkopolska region of Poland assigned in relation to meeting the WHO recommendations of MVPA. We also examined the possible associations between selected environmental and social factors as well as self-evaluation of fitness, appearance, and health status and MVPA after the lockdown. We hypothesised that the decrease in MVPA as a result of this lockdown would be associated with self-evaluating measures and some environmental factors.

## 2. Materials and Methods

### 2.1. Study Design

**Figure 1:**
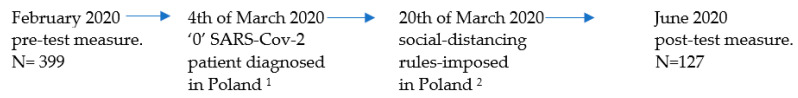
Study design with critical time frames. ^1^ Puls Medycyny, Pierwszy, potwierdzony przypadek COVID-19 w Polsce, https://pulsmedycyny.pl/pierwszy-potwierdzony-przypadek-covid-19-w-polsce-984079 (accessed on 30 November 2020). First confirmed case of COVID-19 in Poland. ^2^ Premier: Wprowadzamy stan epidemii w Polsce, https://www.gov.pl/web/premier/premier-wprowadzamy-stan-epidemii-w-polsce (accessed on 30 November 2020). The Prime Minister’s government imposed a state of epidemic lockdown in Poland.

The study design is a one-group pre-test/post-test over time assessment widely used in behavioral research for the purpose of comparing data from two moments in time. The baseline time (pre-test) was before the implementation of social distancing, and a post-test examination was performed at the end of the first pandemic lockdown of the school system (study period—3 months between March and June 2020) (see Figure 1). The study was originally initiated as a part of another, previous investigation. The research group was selected from a larger study group (*n* = 399), who took part in an ‘in-person’ examination carried out at the end of February 2020 immediately prior to the pandemic in Poland as a part of a cross-sectional study. The original group was composed of six secondary schools from the urban area of the Wielkopolska region (Greater Poland). For this study, a random 33% sample was extracted from the original study group. The sample unit was a school class randomly selected (one class from each secondary school) (see Supplementary Materials). All participants were informed about the study’s aims, risks and benefits, and parental consent was obtained before the study began. Participation in the survey was voluntary, and the total return rate was 85%. The final sample size was *n* = 127. The investigator followed the study to assess the exposure and outcomes over a longer period of time [41]. This approach was aimed at defining the current relationships between the examined concepts in this study.

### 2.2. Participants and Procedure

The study included data collected from 127 students aged M = 15.4 ± 0.5 years (52% girls with a body mass M = 59.43 ± 10.22 kg and height M = 169.98 ± 8.94 cm and 48% boys with a body mass M = 60.22 ± 10.35 kg and height 171.34 ± 8.89 cm) from secondary schools in the Wielkopolska region of Poland. Due to the national restrictions in post-testing, an electronic online survey was provided in the second half of June 2020 (just before the summer holidays) with the use of the same research tools. The questionnaire included enquiries concerning MVPA, an 8-item scale of environmental factors and three subjective questions about health, fitness, and perception of appearance.

During the informed consent process, the responders were assured that all data would be used for research purposes only. Participants’ responses were anonymous and confidential, in accordance with Google’s privacy policy [42]. Furthermore, responders were not allowed to provide any information regarding their contact information details. In addition, participants were able to stop participating in the study and leave at any stage before the end of the process. If they decided to leave the study, their answers would not be saved. The responses were saved simply by clicking on the ‘send’ button provided. Upon completing the survey, participants acknowledged their voluntary consent to participate in this anonymous study. It is worth mentioning that responders were kindly requested to be truthful in their responses.

### 2.3. Physical Activity (MVPA)

PA levels were determined with a screening measure proposed by Prochaska, Salis and Long [43] termed a Physical Activity Screening Measure. The main reason for using this measure was its ease of application in lockdown conditions, when all students were obliged to work at a distance, in a home setting instead of in schools. The reliability was established at ICC = 0.77 and validity at = 0.40. In addition, this measure was used in an earlier population study in Poland [44]. In general, this measure assesses the average number of days per week with at least 60 min spent undertaking various forms of PA during which time, in the participants’ subjective opinion, their heart rate increased, and they experienced a feeling of shortness of breath (higher breathing frequency). Analysed students were requested to respond and mark truthful answer for two questions: Q1. Over the past 7 days, on how many days were you physically active for a total of at least 60 min per day? Q2. Over a typical or usual week, on how many days are you physically active for a total of at least 60 min per day? These questions formed an MVPA index, which was calculated according to the formula originally presented by Prochaska [43]: MVPA = (Q1 + Q2)/2, where MVPA = PA index; Q1= number of physically active days during the past 7 days; Q2= number of physically active days during a typical week. Then, to further analyse the level of undertaken PA, we decided to examine other selected environmental factors that could play a role in meeting or failing to meet the WHO’s recommendation by youths in the context of PA.

### 2.4. 8-Item Environmental Factors’ Scale

In this study, the role of different social and environmental factors influencing engagement in undertaking PA was evaluated with the use of an 8-item scale (lack of classmates, lack of motivation, no direct contact with the PE teacher, possibility of choosing any physical exercise time and possibility of choosing your own exercises, practicing without any criticism and mockery, PE teacher support, home environment) developed specifically for the purpose of the study by the authors themselves. The scale included a question: How do they influence your engagement in PA? This self-report tool was designed to measure the impact of the abovementioned 8-item scale using a five-point Likert scale that allowed the respondents to assess the impact of each particular statement, from 1 (not at all) to 5 (very much), with higher scores corresponding to increased perception. Test–retest reliability values (with a two-week interval) for the same items ranged between 0.66 and 0.88 and allowed us to investigate further the impact of the external environment on the students’ MVPA.

### 2.5. Health Behaviour in School-Aged Children (HBSC)

To measure the declared perception of adolescent health and physical fitness assessment, the selected three (subjective: self-rated health, self-rated fitness, and self-esteem) factors of The Health Behaviour in School-aged Children [45] questionnaire was used in applying the self-reported scoring system.

The tool’s reliability was established using Cronbach’s Alpha test, a measure of internal reliability [46]. The total reliability was 0.88, and the values were 0.81, 0.87 and 0.69, respectively. The scales have acceptable psychometric properties including evidence of construct validity, internal consistency (α = 0.89) and test-retest reliability (r = 0.85). These values indicate good internal consistency for the scoring system as a whole.

### 2.6. Ethics Approval and Consent to Participate

Because this study involved human data, the authors declare that the investigations were carried out following the rules of the Declaration of Helsinki [47]. Conferring to point 23 of the declaration, the study protocol was approved by the Local Bioethics Committee of the University of Medical Sciences in Poznan (decision no. 893/18) before undertaking the research. The personal characteristics of all responders were documented and adequately protected in accordance with standard research ethics.

### 2.7. Statistics

Descriptive statistics were used to define the proportion of responses for each question and the total distribution in the total score of each question. Due to the lack of normal distribution of data, the comparative analysis of differences between the groups (a group of students meeting the recommended PA; and a group of students failing to meet the WHO PA recommendations) was undertaken with the use of Pearson’s chi-squared test. Because some variables were not normally distributed for the further analyses of mean values in the MVPA, the Wilcoxon test was used. For the environmental variables (lack of classmates; lack of motivation; no direct contact with the PE teacher; possibility of choosing any physical exercise time and one’s own exercises; practicing without any criticism and mockery, PE teacher support, home environment), Spearman’s rank correlation coefficients (rs) were used to assess the significance and power of the relationships between variables, with a value of correlation strength: ≤0.39 weak, 0.40–0.59 moderate and ≥0.60 strong [48]. A hierarchical multiple regression was conducted with MVPA score as a dependent variable. The statistical significance was set at *p* ≤ 0.05. All statistical analyses were completed using TIBCO STATISTICA ver. 13.3 (TIBCO software Inc.1984–2017).

## 3. Results

**Figure 2:**
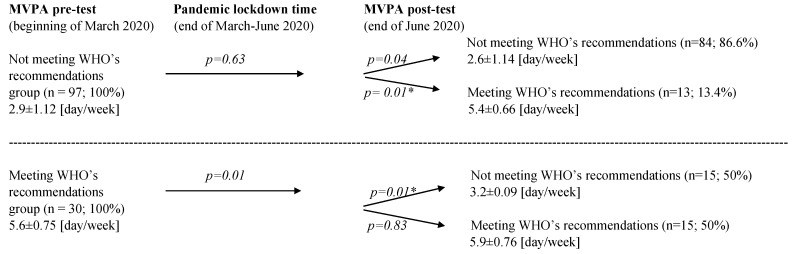
Comparison in meeting WHO’s recommendations for MVPA (days/week) between pandemic pre- and post-test differentiation (M ± SD) within the youth groups. * *p* < 0.05.

A comparative analysis of the entire group of secondary school students (*n* = 127) showed statistically significant differences between pre- and post-test in MVPA (Figure 2).

Data analyses showed a significant difference between pre- and post-test within the group of adolescents who did not meet the WHO’s MVPA recommendations before the lockdown. In post-testing, 13.4% of this original group increased the frequency of PA from 2.6 up to 5.4 [days/week] on average (*p* = 0.01). Majority of the group (86.6%) maintained the same insufficient level of PA per week (*p* = 0.04). In meeting the WHO’s recommendations, the group of students were divided into two subgroups—50% maintained the recommended level (*p* = 0.83), and 50% significantly decreased their level of MVPA below the WHO’s recommendations (*p* = 0.01).

To detect further the impact of the altered PA, Spearman’s rank correlation coefficients (rs) were used (Table 1) between the selected independent variables and MVPA.

Based on the correlation method used, in most cases, no statistically significant relationships were found between pairs of variables (Table 1), and only few variables concerning different sources of self-rated behaviours and social relationships showed a significant correlation with MVPA. Self-rated health for PA showed a significantly strong correlation with MVPA in the group of students who met the WHO recommendations before the pandemic (r = 0.76; *p* < 0.05) but failed to maintain that recommendation during the COVID-19 restrictions. Significant-moderate correlation with MVPA (r = 0.42; *p* < 0.05) was observed in the group of students who did not meet the WHO recommendations before pandemic, and maintained the unsatisfactory level of MVPA during lockdown. A significant but weak correlation was observed between MVPA and lack of motivation among those who failed in WHO recommendations in both pre- and post-tests (r= −0.27). In the same group, a medium correlation (r = 0.41; *p* < 0.05) was found between the possibility of choosing exercise time and MVPA, and a weak correlation was observed for the possibility of choosing exercises and MVPA (r = 0.28; *p* < 0.05). The only significant but moderate correlation was noted (r = 0.56; *p* < 0.05) in the group of meeting WHO recommendations in the COVID-19 situation with the no criticism or mockery in practice factor.

Because the correlation analyses did not explain the role of the selected factors on MVPA, hierarchical multiple regression as a further research step was conducted. Hence, stepwise regression was performed separately for the group not fulfilling WHO recommendations during the pandemic and for those fulfilling these recommendations. In the group that failed to meet the WHO recommendations, the regression model included four variables that correlated with MVPA but only one significantly (see Table 2).

In the regression model for changes in MVPA, the relationships within self-rated fitness (*p* = 0.022) were the first to enter the statistically significant (*p* < 0. 05) regression equation. The model explained 10% of the variance of the dependent variable (adjusted R^2^ = 0.10) (Table 3). It was well matched to the data—better than the average—and predicted a dependent variable.

## 4. Discussion

The aim of this study was to analyze the impact of lockdown (which brought in the regulations of social distancing during the period of the first wave of COVID-19) on the level of PA in Polish youth. The obtained research results turned out to be interesting from a public health point of view. Considering this research, we can already assess the impact of restrictions on the health-related behaviours (e.g., PA). Our findings are in line with some other studies on the impact of the COVID-19 pandemics on the active lifestyle of adolescent youth [2,12,30], but since this issue is relatively new more studies may help in getting a broader picture from various contexts, here specifically in a school setting.

Even before the pandemic, there was strong evidence that most children and adolescents (80% of 1.6 million school-going adolescents aged 11–17 years) in many countries do not participate in sufficient PA [13,49]. The imposed restrictions have negatively affected several life domains among young people; in particular, youths reported reduced health-related quality of life [50], negative psychological and emotional outcomes [51,52,53], behavioural difficulties [54], poor nutritional regimen [55] and increased sedentariness and screen time [56,57]. We found too that the pandemic led to a 50.4% PA decrease of those surveyed from 4.0 to 2.5 days per week with sufficient PA. In 38.6% of students, an increase in PA was observed from 3.0 to 4.5 days a week, whereas 11% of students remained at the same level of PA (3 days/week). These results are similar to the findings among the Irish population of adolescents [58], in which less PA was related to people with overweight or who were obese. Xiang [57] also observed a significant reduction in PA and an increase in TV watch time during the COVID-19 pandemic while studying PA levels of 2426 children and adolescents in March 2020 in China. The median time spent in PA decreased drastically, from 540 min/week (before the pandemic) to 105 min/week (during the pandemic), yielding a 435-min reduction on average [57]. The authors noted that during the pandemic, the incidence of physically inactive students increased significantly from 21.3% to 65.6%, and screen time increased (on average plus 1730 min, or approximately 30 h, per week) [57]. Thus, the above research results confirm both our hypothesis and analyses (albeit on a small group), demonstrating the negative impact of the pandemic on PA undertaken by young people, and Hall’s conclusions [59] that we are currently confronted with two pandemics occurring concurrently: COVID-19 and the pandemic that has involved physical inactivity (PI) and sedentary behaviours (SB) since 2012 and, more worryingly, that we may be at risk of exacerbating this second pandemic as a result of the first.

Limited PA and an inability to take a regular walk out of one’s home as a consequence of collective quarantine may be associated with several metabolic effects [60]. It is established that many beneficial metabolic and cardiovascular adjustments in response to physical exercise can be lost in only two weeks’ time of inactivity, impairing aerobic capacity and/or increasing blood pressure [61]. Sudden exercise cessation has been associated with the rapid onset of insulin resistance in muscle tissue and decreased muscle glucose utilisation that led to worsened muscle performance [62]. These observations indirectly explain our findings that physical fitness as a result of a sudden decrease in PA was the most frequently quoted variable that changed during the pandemic. The strength of this relationship was dependent upon the group being analysed. The strongest correlation, however, was noted in the group that met the WHO’s recommendations before the collective quarantine.

For adolescents, social interactions and friendships significantly influence PA behaviours [63]. In turn, engaging in physical exercise and fitness in adolescence may be appropriate for improving health and well-being, contributing to better development at this stage. Reigal and colleagues’ [64] findings indicate that adolescents who practiced more weekly physical exercise and had a higher level of physical fitness scored better on the cognitive functioning and psychosocial tests evaluated. Frömel et al. [65] provided similar results, demonstrating that the least depressive symptoms (DS) and the highest well-being were observed in boys and girls with high PA levels in the dimension of recreational PA, which has been highly limited due to sanitary restrictions. In turn, Pinto et al. [66] found PI and not participating in PE classes increased loneliness, and SB and PI were associated with anxiety and DS [67]. Strong, consistent evidence was found for the relationship between both DS and leisure screen time, particularly when that screen time accounts for more than 2 or 3 h per day [68].

Given the above findings, staying active and maintaining a physical exercise routine seems to be essential for mental and physical health [69]. The Internet could prove useful in this context for following online exercise and using video- or app-guided aerobics training at home. Although it is still under debate whether PA is better accomplished indoors or outdoors, it seems clear that the amount of social contact and support from others increases the beneficial effects of PA interventions [70]. It can be advisable to increase the PA intensity to enhance the exercise effect on mental health [71,72]. However, for reasons of stimulus effectiveness, every factor of PA prescription (e.g., volume, intensity) should be individually tailored and monitored [73,74], thus, professional advice is always desirable.

Moreover, we have established that in the group who had not met WHO’s recommendations before the lockdown, some participants significantly increased their PA. As found by Hargreaves et al. [75] in their study in New Zealand, vigorous and moderate intensity PA were significantly lower post-lockdown compared to pre-lockdown in those individuals who had been highly active pre-lockdown with simultaneous contrast for moderately active individuals’ pre-lockdown, and vigorous and moderate intensity PA was significantly higher during-lockdown compared to pre-lockdown [75]. Our PA results show how important it is to analyze the PA data collected during COVID-19 restrictions depending on how active the adolescents were before the restrictions as the pattern of change was different between the groups meeting and not meeting WHO’s recommendations. Given that most of the research published so far examining PA during COVID-19 restrictions has been quantitative, the explanations for why PA has changed have not been thoroughly explored. We can therefore only presume the determinants of this change. According to Hargreaves et al. [75] 35% of participants who increased or maintained their PA levels during lockdown noted that being active “was a good excuse or reason to get outside”. Young people may also want to increase their physical fitness or just have more free time to use it for this purpose. Alternatively, moderately active individuals, compared to highly active individuals, may not have needed access to sport facilities to significantly increase their PA levels. Being active close to home during-lockdown via simple modes of PA such as running and cycling, or at home using exercise equipment and online exercise videos (which was common during lockdown) [76], may have been attractive for these moderately active individuals. It could also be due to autonomic motivation, which is a strong predictor of PA behavior [77]. Some evidence for this explanation was provided by the participants of the study by Hargreaves et al. [75], who, when asked “why were you more active / continued to be active from period to post-closure”, stated that it was important for physical and mental well-being. The study had several limitations that should be mentioned. The relatively small sample size could be considered a limitation, although random selection of the examined individuals might counteract this issue. Our data were mostly self-reported, which is susceptible to cognitive bias, so in the further research device-measured methods in assessment of the level of PA should be consider. Furthermore, repeated measurements (pre/post-test design) strengthened the study but certainly did not allow for a population-specific generalisation of the changes in that period. It might also be the case that in some other countries, social or cultural background issues could provide different outcomes. Nevertheless, the impact of a nation-wide lockdown on PA levels of young people is worthy of more in-depth investigation with studies of various designs considering longitudinal studies as the way how to define the causality and determinants of PA. Additionally, such studies should consider the year-round movement behaviours concerning different lifestyle patterns.

## 5. Conclusions

The COVID-19 restrictions at local and national levels and the resulting impact on physical and mental health status have consequences for post-COVID-19 in both the short- and long-term perspective. PA might be a key factor to assist the population to better tolerate pandemic periods at both physical and mental levels. Therefore, these two factors should be considered as being effective in lessening the impact of these times and should be included in general health care and prevention strategies from local up to global dimensions.

The results suggest the need for increased actions on the part of both PE teachers and parents in the context of motivating and supporting young people in taking up conscious PA in order to prevent any threat to the population’s health, specifically in lockdown situations.

The results may also indicate a low level of awareness of PA and a sense of the need for implementing it without external control among young adolescents, particularly because adolescents’ physical literacy could help build an internal system of PA needs [78]. The research results have major implications for government and educational-based support initiatives in planning assistance for the young population in case of possible future long-time lockdown situation when particular attention should be paid to PA.

Future research should explore whether the observed impacts on PA of youths and social support levels remain, worsen, or diminish (via adjustment) as the pandemic continues and how this development is resolved in the long-term perspective.

## Figures and Tables

**Table 1:** Correlation among study variables.

	*Pre-Test* *Not Meeting WHO Recommendations*		*Pre-Test* *Meeting WHO Recommendations*	
*Variables*	Post-Test Not Meeting WHO’s Recommendations	Post-Test Meeting WHO’s Recommendations	Post-Test Not Meeting WHO’s Recommendations	Post Test Meeting WHO’s Recommendations
*Self-esteem*	−0.00	0.07	0.05	0.10
*Fitness*	0.03	−0.25	0.48	0.12
*Health*	0.42 *^,2^	0.03	0.76 *^,3^	0.34
*Lack of peers*	0.16	0.47	0.19	0.16
*Lack of motivation*	−0.27 *^,1^	0.43	−0.49	−0.21
*No direct contact with PE teacher*	0.01	0.10	0.27	0.28
*Possibility of choosing exercise time*	0.41 *^,2^	0.23	0.08	0.14
*Possibility of choosing exercises*	0.28 *^,1^	0.04	0.02	−0.00
*No criticism, mockery in practice*	0.16	0.56 *^,2^	−0.13	−0.12
*PE teacher support*	−0.03	−0.21	0.26	−0.51
*Home environment*	0.16	−0.34	−0.06	0.38

*—*p* < 0.05; correlation ≤ 0.39 weak ^1^, 0.40–0.59 moderate ^2^, and ≥0.60 strong ^3^.

**Table 2:** Summary of dependent variable MVPA in adolescents, identified on hierarchical regression analysis.

MVPA	Β	SE	*p-Value*
*Self-rated Fitness*	0.32	0.14	0.022
*Home environment*	−0.13	0.09	0.138
*Lack of motivation*	0.13	0.08	0.107
*Lack of classmates*	−0.08	0.07	0.289
	R = 0.299 R^2^ = 0.051 F(4.94) = 2.3241 *p* < 0.062		

**Table 3:** Summary of dependent variable MVPA in adolescents, identified on hierarchical regression analysis.

MVPA	B	SE	*p-Value*
*Self-rated Fitness*	0.03	0.15	0.058
	R = 0.361 R^2^ = 0.097 F(1.26) = 3.9070 *p* < 0.058		

## Data Availability

The data presented in this study are available on request from the corresponding author. The data are not publicly available due to no access to publicly accessible repository.

## Supplementary Materials

The following are available online at https://www.mdpi.com/article/10.3390/ijerph18105139/s1. COVID całość 2020 wersja ostateczna (the final version of COVID 2020).
